# A Mouse Model for the Rapid and Binomial Assessment of Putative WNT/β-Catenin Signalling Inhibitors

**DOI:** 10.3390/biomedicines11102719

**Published:** 2023-10-07

**Authors:** Janson Tse, Ryan O’Keefe, Angela Rigopolous, Annalisa L. E. Carli, Jo Waaler, Stefan Krauss, Matthias Ernst, Michael Buchert

**Affiliations:** 1 Cancer and Inflammation Laboratory, Olivia Newton-John Cancer Research Institute, Heidelberg, VIC 3084, Australiamatthias.ernst@onjcri.org.au (M.E.); 2 School of Cancer Medicine, La Trobe University, Bundoora, VIC 3086, Australia; 3 Tumour Targeting Laboratory, Olivia Newton-John Cancer Research Institute, Heidelberg, VIC 3084, Australia; 4 Department of Immunology and Transfusion Medicine, Oslo University Hospital, Rikshospitalet, 0424 Oslo, Norway; 5 Hybrid Technology Hub-Centre of Excellence, Institute of Basic Medical Sciences, Faculty of Medicine, University of Oslo, 0317 Oslo, Norway

**Keywords:** WNT/β-catenin signalling, WNT inhibitors, in vivo assay, tankyrase inhibitor, pyrvinium pamoate, *APC*

## Abstract

Specific signalling thresholds of the WNT/β-catenin pathway affect embryogenesis and tissue homeostasis in the adult, with mutations in this pathway frequently occurring in cancer. Excessive WNT/β-catenin activity inhibits murine anterior development associated with embryonic lethality and accounts for the driver event in 80% of human colorectal cancers. Uncontrolled WNT/β-catenin signalling arises primarily from impairment mutation in the tumour suppressor gene *APC* that otherwise prevents prolonged stabilisation of β-catenin. Surprisingly, no inhibitor compounds for WNT/β-catenin signalling have reached clinical use in part owing to the lack of specific in vivo assays that discriminate between on-target activities and dose-limiting toxicities. Here, we present a simple in vivo assay with a binary outcome whereby the administration of candidate compounds to pregnant and phenotypically normal *Apc^flox/flox^* mice can rescue in utero death of *Apc^min/flox^* mutant conceptus without subsequent post-mortem assessment of WNT/β-catenin signalling. Indeed, the phenotypic plasticity of born *Apc^min/flox^* conceptus enables future refinement of our assay to potentially enable dosage finding and cross-compound comparisons. Thus, we show for the first time the suitability of endogenous WNT/β-catenin signalling during embryonic development to provide an unambiguous and sensitive mammalian in vivo model to assess the efficacy and bioavailability of potential WNT/β-catenin antagonists.

## 1. Introduction

The canonical WNT/β-catenin signalling pathway provides the core of an evolutionary conserved, fundamental pathway that controls growth and governs numerous developmental processes, while its aberrant regulation contributes to many diseases including cancer [1,2,3]. Despite more than two decades of development to identify WNT/β-catenin signalling inhibitors [4,5,6], including G007-LK-series tankyrase inhibitors [7,8,9,10], no drug has so far reached clinical use. This is in part due to the lack of suitable whole animal models that allow for the rapid and unambiguous monitoring of on-target activity, while simultaneously predicting side effects that may arise from the broad and iterative use of the WNT/β-catenin signalling pathway for homeostatic tissue renewal throughout adult life [11].

The canonical WNT/β-catenin signalling pathway is activated by the interaction of WNT ligands with their cognate frizzled (FZD) receptors at the cell surface. Upon binding of WNT ligands to FZD, cytoplasmic AXIN proteins associate with phosphorylated lipoprotein receptor-related protein (LRP) to deactivate the adenomatous polyposis coli (APC)/AXIN destruction complex, leading to the stabilisation of β-catenin through phosphorylation and its subsequent translocation to the nucleus where it binds to T-cell factor (TCF) family members to upregulate target genes [12]. Under homeostatic conditions, the activity of the WNT pathway is tightly controlled by the destruction complex, which continually degrades cytoplasmic β-catenin thus preventing its nuclear translocation. Mutations that inactivate the function of the destruction complex or stabilise β-catenin result in WNT ligand-independent activation of the canonical WNT signalling cascade and such mutations are frequently observed in cancer [13,14]. For example, more than 80% of sporadic colorectal cancer (CRC) in humans harbour impairment or null mutations in the *APC* gene, leading to uncontrolled stabilisation of β-catenin and the formation of constitutive complexes between β-catenin and members of the TCF family [15,16].

In the mouse embryo, the WNT/β-catenin signalling pathway is essential for the establishment of the anterior-posterior axis during gastrulation and for the development of the neural tube, somites, and limbs [17,18]. Thus, activation of this pathway promotes anteriorisation, while pathway inhibition can result in posteriorisation of the embryo [18], and mutations in components of the canonical WNT pathway result in developmental abnormalities, congenital malformations, all the way to embryonic lethality [19]. Canonical WNT signalling also plays a role during the formation of the heart and many other aspects of organogenesis [20]. Notably, during eye development, this pathway contributes to eye field specification, optic cup and lens morphogenesis, and retinal cell differentiation [21].

In the present study, we harnessed genetic mouse models with various activation levels of canonical WNT signalling using two different hypomorphic *Apc* mutant alleles, *Apc^flox^* and *Apc^min^*. In its naïve, unrecombined state, the *Apc^flox^* allele harbours two loxP recombination sites flanking exon 14 of the *Apc* gene, thereby rendering this allele transcriptionally active albeit producing reduced expression of full-length 2843 amino acid APC protein [22]. Meanwhile, the *Apc^min^* allele encodes a nonsense mutation at codon 850, resulting in the expression of a non-functional, truncated APC protein that is unable to bind to β-catenin and therefore prevents effective degradation of β-catenin [23]. While both mutations individually increase the level of canonical WNT signalling, in vivo, embryonic, and postnatal life remains unaffected. In contrast, concurrent expression of both alleles generates a fully penetrant embryonic lethal phenotype characterised by the absence of anterior head structures, which is completely prevented in *Apc^min/flox^;ctnnb1^+/−^* offspring [24]. This observation suggests that the reversal of the development defects of *Apc^min/flox^* embryos could serve as a bimodal readout for a low-throughput screening platform to evaluate the specificity and biological availability of antagonists for canonical WNT signalling. Here, we provide proof-of-principle evidence for the validity of this hypothesis by showing that timed administration of WNT pathway inhibitors during pregnancy rescues embryonic lethality and anterior specification in *Apc^min/flox^* embryos.

## 2. Materials and Methods

### 2.1. Study Approval

All mouse experiments were carried out in compliance with every pertinent ethical regulation for animal research and were authorised by the local Animal Ethics Committee of Austin Health.

### 2.2. Animal Models

*Apc^min/+^* and *Apc^flox/flox^* mice have been previously described [22,23] and were bred and housed under specific pathogen-free conditions in the Biological Resources Facility at Austin Health on a 12 h light/dark cycle at a fixed temperature with free access to standard chow and drinking water. All experiments were conducted during the light cycle. Matings were set up overnight, and the presence of a vaginal mating plug the next morning was considered proof of successful coupling. Mated females were then separated and observed for signs of pregnancy (weight gain, nesting behaviour, abdominal bulge).

### 2.3. Animal Treatments

The tankyrase inhibitor G007-LK was administered to the diet by providing commercially prepared feed pellets (Speciality Feeds, Glen Forrest, Australia) at an inclusion rate of 600 mg per kg chow. The daily food intake of individually housed mice is about 15–20 g per 100 g body weight [25], which equates to approximately 2.4 mg of G007-LK/day/mouse. Pyrvinium pamoate (USP Rockville, MD, USA) was prepared at 5 mg/mL in HPMC-SV ((0.5% *w*/*v*) hydroxypropyl methylcellulose, 0.5% (*v*/*v*) benzyl alcohol, 0.4% (*v*/*v*) Tween 80) and given at 25 mg/kg by oral gavage.

### 2.4. Immunohistochemistry

The formalin-fixed and paraffin-embedded tissue samples were cleared in two successive xylene baths for 10 min each before being rehydrated in tiered ethanol baths (70%, 100%). Tissue antigen retrieval was performed in sodium citrate buffer (10 mM, pH 6.0) or EDTA buffer at 95 °C for 18 min. Tissue sections were then incubated in 3% H_2_O_2_ in PBS for 20 min at RT to block endogenous peroxidases followed by immersion in normal goat serum (NGS, 5%) for 1 h at RT to block nonspecific binding sites. Tissue sections were stained with various primary antibodies (anti-CPS, Ab3682, 1:1000, Abcam; anti-Cyp2e1, PA5-35351, 1:500, Invitrogen, Waltham, MA, USA) in blocking buffer for 16 h at 4 °C. Excess antibody was removed by washing twice with 1× PBS/0.1% Tween and once with 1× PBS for a total of 30 min followed by incubation with secondary antibodies at RT for an additional 30 min. After three washes in 1× PBS, the sections were immersed in diluted (1:200 PBS) ABC solution (Vectastain, ABC-Kit, Vector Laboratories, Newark, CA, USA) at RT for 30 min. Staining was developed by adding DAB to tissue sections, and the staining process was stopped by adding distilled water to the sections. Hematoxylin was used for counterstaining of tissue sections followed by incubation in ethanol gradients (100%, 70%) and xylene for dehydration. Tissue sections were set in mounting media.

### 2.5. Real-Time PCR

Total RNA was prepared from small intestines using Trizol^®^ Reagent (Life Technologies, Carlsbad, CA, USA), and 2 µg of total RNA was used as input for cDNA preparation with the high-capacity cDNA reverse transcription kit (Applied Biosystems, Waltham, MA, USA) as per the manufacturer’s instructions. Quantitative (q)RT-qPCR analysis was conducted using the SensiMix SYBR kit (Bioline, London, UK) in technical triplicates on the ViiA 7 Real-Time PCR system (Life Technologies, Carlsbad, CA, USA).

### 2.6. T2 MRI Scan

Magnetic Resonance Imaging (MRI) acquisition was performed using a T2-weighted sequence using a 2D Fast Spin Echo sequence (FSE). The parameters for 2D FSE are slice thickness = 1 mm, resolution= 0.21 × 0.21 mm^2^, 25 slices, and number of excitations = 2. Mice were imaged with a small animal camera (nanoScan^®^ PET/MRI camera, Mediso, Budapest, Hungary).

### 2.7. Statistical Analysis

Statistical analyses were carried out in Prism8 (GraphPad, San Diego, CA, USA). Statistical significance was assessed by two-tailed unpaired *t* tests, and 2 × 2 contingency tables were calculated using Fisher’s exact test. Sample size calculation was carried out using the sample size calculator at ClinCalc.com (https://clincalc.com/stats/samplesize.aspx, accessed on 26 April 2023). Data are expressed as mean ± standard error of the mean.

## 3. Results

### 3.1. Administration of TNKSi Attenuates WNT Pathway Activity In Vivo and Partially Rescues Cranial Defects In Vivo

WNT pathway inhibitors are a commodity in high demand owing to the large number of pathologies associated with aberrant, or excessive WNT/β-catenin signalling. In order to develop a sensitive assay with an unambiguous in vivo efficacy and toxicity readout for WNT pathway inhibitors, we made use of our previous observations that murine *Apc^min/flox^* embryos fail to develop anterior head structures from E10.5 onwards and subsequently die in utero at E15. Because this phenotype is prevented by monoallelic ablation β-catenin in corresponding *Apc^min/flox^* embryos [24], we hypothesized that the administration of WNT pathway inhibitors to pregnant females would restore physiological anterior/posterior development in *Apc^min/flox^* embryos and overcome the associated embryonic lethality. Hence, we set up timed matings between female *Apc^flox/flox^* with male *Apc^min/+^* mice to yield equal ratios of *Apc^min/flox^* and *Apc^flox/+^* conceptions, with only the *Apc^flox/+^* embryos developing to term.

As an initial proof-of-concept, we treated pregnant mothers with the preclinical tankyrase 1/2 inhibitor (TNKSi) G007-LK [8,10]. This compound has shown on-target activity in vivo by restricting the proliferation of Lgr5+ stem cells in the murine intestine [26]. In light of our observations that subtle genetic exaggeration of β-catenin increased intestinal tumour formation in *Apc^min/+^* mice without loss-of-heterozygosity of the wild-type *Apc* allele, and that monoallelic ablation of β-catenin rescued the anterior defect in *Apc^min/flox^* embryos [24,27], we established treatment conditions where G007-LK would only confer partial effects. Thus, we confirmed that G007-LK, administered through inclusion in chow and consumed at a rate of 120 mg/kg/day, could inhibit the expression of bona fide target genes of the canonical WNT/β-catenin pathway in the intestinal epithelium of *Apc^flox/flox^* mice. When administered as G007-LK-containing chow over three consecutive days immediately prior to RNA expression analysis of the intestinal epithelium, this treatment reduced expression by 50–70% across *Axin2*, *Sox9*, *Cd44*, and *Ccnd1* (Figure S1A–D).

In light of the importance of the WNT/β-catenin pathway during gastrulation and many other subsequent stages of embryonic development [24,28,29,30,31,32], we reasoned that administration of a WNT inhibitor during the entire duration of pregnancy was likely to result in the abortion of the foetus. Thus, we set out to establish a treatment window during which acute administration of G007-LK-containing chow would suppress aberrant posteriorisation in *Apc^min^*^/*flox*^ embryos.

We previously detected the earliest signs of elevated WNT signalling in E3.5–5.5 *Apc^min^*^/*flox*^ embryos at the anterior border of the embryonic visceral endoderm [24]. However, our attempts at treating pregnant *Apc^flox^*^/*flox*^ mice between E2–3 and E4–7 resulted in disrupted embryonic development as judged by the failure of pregnant mice to gain weight and develop an abdominal bulge despite the presence of a vaginal mating plug. We made a similar observation when G007-LK-containing chow was administered to “plugged” females during the corresponding E5–14 and E5–10 stages of embryonic development. Meanwhile, the administration of G007-LK-containing chow during the E5–6 stages yielded deformed and non-viable embryos at E15 that underwent subsequent spontaneous abortion (Table 1).

**Figure 1:**
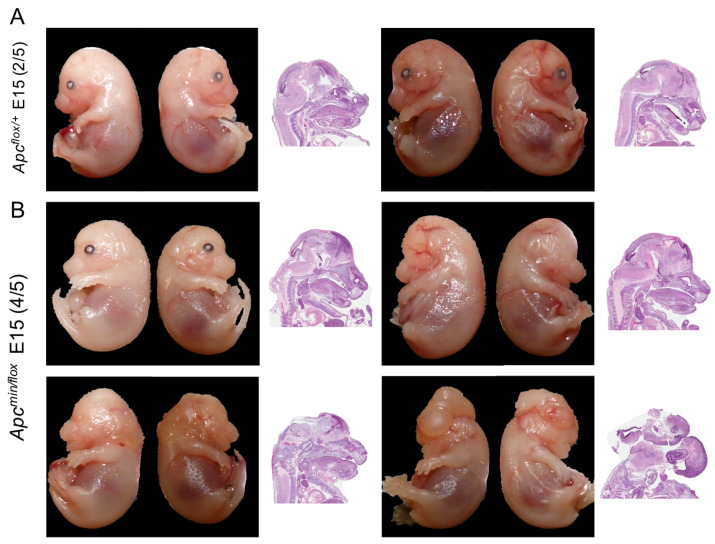
In vivo administration of TNKSi partially rescues cranial abnormalities. E15 embryos extracted from *Apc^flox/flox^* mice impregnated by *Apc^min/+^* male mice after treatment with G007-LK at embryonic days 5–7. Pictures were taken after extraction with accompanying H&E staining of the sagittal section to observe cranial features. Images show representative embryos of the litter consisting of 5 *Apc^flox/+^* (**A**) and 5 *Apc^min/flox^* (**B**) embryos. Of the 5 *Apc^min/flox^* mice, 2 had the same appearance as the *Apc^flox/+^* mice.

Indeed, expanding the treatment by one more day, i.e., during E5–E7, enabled extension of in utero development of *Apc^min^*^/*flox*^ embryos past E15. Accordingly, in a prototypic litter of E15 embryos, we found the expected 1:1 Mendelian distribution between *Apc^min^*^/*flox*^ and *Apc^flox^*^/+^ embryos. Furthermore, of the five *Apc^min^*^/*flox*^ embryos, two appeared phenotypically indistinguishable from their *Apc^flox^*^/+^ littermates. Meanwhile, three *Apc^min^*^/*flox*^ embryos harboured cranial abnormalities with different degrees of posterior abnormality, including one *Apc^min^*^/*flox*^ E15 embryo with a complete head structure but lacking both eyes (bilateral anophthalmia; Figure 1A,B; Figure S1E,F). Collectively, these anecdotal observations suggested a very tight but reproducible treatment window during which administration of WNT signalling inhibitor to pregnant mothers during E5–E7 of embryonic development may result in the birth of live *Apc^min^*^/*flox*^ pups.

### 3.2. In Vivo Administration of TNKSi Results in Live Births of Apc^min/flox^ Mice

**Figure 2:**
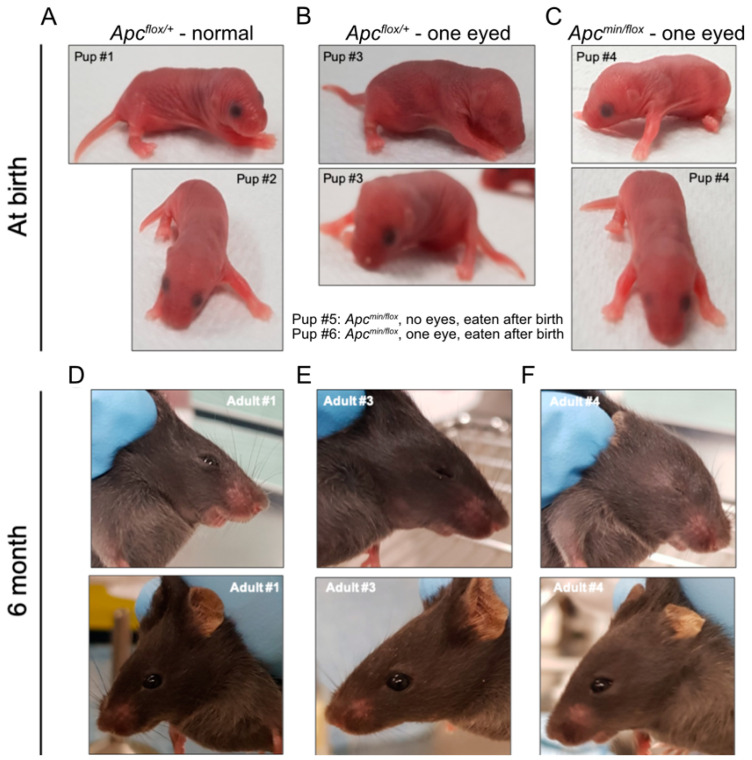
In vivo administration of TNKSi results in live births of *Apc^min/flox^* mice. Pups born alive from *Apc^flox/fl^*^ox^ mice impregnated by *Apc^min/+^* male mice after treatment with TNKSi at embryonic days 5–7. Pictures were taken on the day of birth (**A**–**C**) and at 6 months of age (**D**–**F**). Genotype and eye phenotype are as indicated. # *indicate individual new-born and adult mice*.

**Figure 3:**
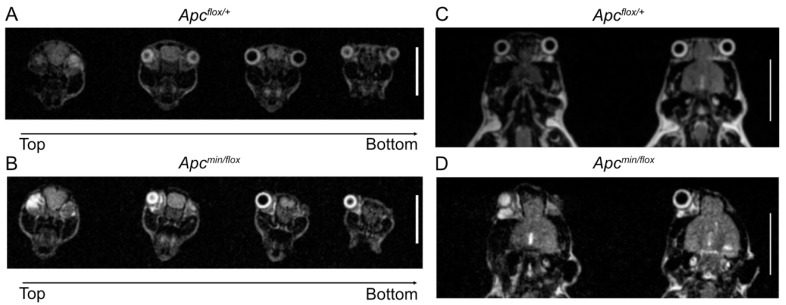
Development of cranial features in treated *Apc^flox/+^* and *Apc^min/flox^* mice. T2 MRI head scans of 6-month-old *Apc^flox/+^* and *Apc^min/flox^* mice taken from an axial (**A**,**B**) and coronal view (**C**,**D**). Each image in the axial plane represents a slice taken from the top of the head to the bottom. Scale bar = 1 cm.

To test the above hypothesis, we treated several time-mated pregnant *Apc^flox^*^/*flox*^ females with G007-LK-containing chow and recovered sixteen live pups at birth and another three stillborns across three independent litters. Half (8) of the live pups and all the three stillborn pups returned *Apc^min^*^/*flox*^ genotypes. Strikingly, all stillborn pups had cranial defects, while all live *Apc^min^*^/*flox*^ pups had anophthalmia, including five with bilateral anophthalmia and three with a monolateral phenotype (Figure 2A–C; Table 2). Interestingly, monolateral anophthalmia was also observed in one of the eight *Apc^flox^*^/+^ pups. While we observed that pups affected by anophthalmia became cannibalized within the first 24 h after birth (Table 2), our follow-up studies on a small cohort of *Apc^min^*^/*flox*^ mice with unilateral anophthalmia suggested normal developmental processes towards adulthood without any additional life-threatening pathologies (Figure 2D–F). Indeed, MRI scans of 6-month-old *Apc^flox^*^/+^ and *Apc^min^*^/*flox*^ littermates affected by anophthalmia revealed no discernible differences between their cranial structures (Figure 3A–D).

To cross-validate our in vivo model with a second putative WNT signalling antagonist, we focused on pyrvinium pamoate, which antagonises WNT signalling through the stimulation of CK1α activity [33] and can suppress intestinal WNT signalling in vivo [34]. Adhering to the treatment window established with G007-LK, we administered 25 mg/kg pyrvinium pamoate p.o. to *Apc^flox^*^/*flox*^ females during stages E5–E7 of their pregnancies. In a representative litter, we found two pups born alive, including one with monolateral anophthalmia, and two stillborn pups with cranial defects. Genotyping revealed that all, except the phenotypically normal pup, were *Apc^min^*^/*flox*^ (Figure S2A–D; Table 2).

Taken together, our data establishes and validates a novel, rapid, and reproducible in vivo test system for the activity assessment of putative WNT signalling inhibitors based on unambiguous binomial outcomes.

## 4. Discussion

The multitude of biological processes and diseases that underlie de-regulated canonical WNT signalling make this pathway an obvious candidate for drug development. On the flip side, inhibition of the pathway may cause on-target toxicity and major clinical concerns, due to the central role of canonical WNT signalling as a marshal of developmental and regenerative processes, as well as a regulator of epithelial stem cell compartments and associated maintenance of tissue homeostasis [35]. Our results address both aspects by establishing an in vivo model suitable for testing putative WNT signalling antagonists and assessing WNT signalling threshold levels required to enable physiological processes and protect against cancer and other pathologies. Our model provides an assay for a physiological readout building on the hypomorphic function of an *Apc^flox^* allele, where a neomycin expression cassette in intron 13 interferes with the un-incumbent transcription and associated expression of naïve *Apc* [22]. Because of the function of APC as a rate-limiting component of the β-catenin destruction complex that retains canonical WNT signalling within physiological limits, the naïve *Apc^flox^* allele reduces the capacity to buffer excessive β-catenin activity. Thus, the resulting *Apc^flox/+^* cells become sensitised so that additional monoallelic *Apc* mutations fail to provide threshold barriers for WNT signalling, which enables normal developmental processes without triggering pathologies.

The consistent return of live *Apc^min/flox^* pups affected by anophthalmia following treatment with WNT antagonists strongly argues against the need for genotyping of pups in order to confirm the activity of a putative WNT inhibitor. While anophthalmia occurs in wildtype C57BL/6 mice at an incidence rate of less than 4.3% [36,37], this rate increased to > 55% (10/18) in litters born from WNT inhibitor-treated pregnant *Apc^flox/flox^* mice without further assignment of the pups to one of the two genotypes (i.e., *Apc^min/flox^* and *Apc^flox/+^*). This increase is statistically highly significant when compared to the background rate of anophthalmia in either wild-type or litters of treatment-naïve *Apc^flox/flox^* mice (Table 3, Fisher’s exact test). Thus, designing in vivo inhibitor treatment tests as studies with two independent arms (i.e., treatment vs. control) and with anophthalmia-positive/-negative as a binomial endpoint will require an assessment of *n* > 11 pups to detect a treatment effect, based on a power of 80% and a probability of type I error of 0.05 (Table 4). Accordingly, this vastly simplifies the use of this model as a low throughput in vivo screen.

Here, we show that in utero development of *Apc^min/flox^* conceptus affords an in vivo testing assay with an unambiguous binomial “read-out” for the activity of putative WNT inhibitors. This result is obtained within two weeks of compound administration and does not require any form of specialized post-mortem tissue analysis. Although our model requires that candidate molecules be transported across the placenta, we note that most small molecules can freely transfer through the placenta [38]. Moreover, the spectrum of phenotypes across *Apc^min/flox^* pups born (i.e., still-born, extent of anophthalmia in live mice) suggests that our model could be further refined to also provide insights into dose-activity relationships for putative inhibitors. Our data presented here provide striking evidence that our system faithfully records the activity of inhibitors that functionally act down-stream of Apc in the canonical WNT signalling cascade. However, we have previously found that *Apc^min/flox^* cells remained responsive to WNT ligands, implying that the “rescue” of the developmental defects in *Apc^min/flox^* embryos may potentially also result from interference with WNT signalling up-stream of Apc.

In addition to establishing and validating a novel in utero assay for testing the activity of inhibitors of canonical WNT signalling, the “rescued” *Apc^min/flox^* mice here provide preliminary biological insights into the relationship between specific biological processes and the associated signalling thresholds. For instance, ectopic activation of WNT/β-catenin signalling in the lens placode prevents lens specification, and this is associated with deficiency of the Paired Box 6 (Pax6) gene [39,40,41]. Interestingly, expression of a stabilised β-catenin isoform under the control of Pax6 enhancer elements confers severity on lens development across littermates [41], which is reminiscent of the anophthalmia variability across in the WNT inhibitor-rescued *Apc^min/flox^* pups. We also observed expansion of the WNT target gene Cyp2e1 and the loss of periportal maker carbamoyl-phosphate synthase in 6-month-old *Apc^min/flox^* mice (Figure S3A), suggesting that elevated hepatic β-catenin signalling affects zonation to favour expansion of pericentral at the expense of a periportal expression program [24,42].

Comparing phenotypes across allelic series identifies threshold levels between physiological and pathological consequences, including the anterior–posterior axis formation discussed here. We have previously ranked the three genotypes discussed here with respect to responding to the WNT3a ligand, suggesting the lowest activity in *Apc^flox/flox^* and the highest in *Apc^min/flox^* cells, with the activity in *Apc^min/+^* cells being intermediate [24]. Interestingly, intestinal tumours and associated splenomegaly occur significantly later in *Apc^min/flox^* mice than in *Apc^min/+^* mice (6 mo vs. <3 mo; Figure S3B,C) because of somatic loss of the remaining wild-type allele. Thus, we place the signalling threshold underpinning a reproducible occurrence of this phenotype to be above that of the *Apc^min/flox^* allele combination. By contrast, the signalling threshold enabling hepatocellular carcinomas is below that of *Apc^flox/flox^* cells, since HCC occurs in the latter mice at 18 months of age [24] and notably 6–9 months after *Apc^min/+^* mice succumbed to intestinal tumours. Meanwhile, complete inactivation of Apc in the livers of *TTR^Cre^*; *Apc^flox/flox^* mice results in severe hyperammonemia and hyperglutaminemia due to changes of metabolic liver zonation changes resulting in the rapid onset of fatality [43]. Thus, analogous to the example for Apc discussed here, allelic series for other signalling molecules can provide both the basis for the development of whole animal-based drug structure-activity assays and provide insights into how specificity can be imposed across pathways with pleiotropic activity.

Collectively, our findings here further support the existence of tissue and development-specific ‘just-right’ WNT signalling activation thresholds that demarcate between responses compatible with tissue homeostasis and those required for regenerative processes and/or underlying the development and progression of disease (Figure S4). A better understanding of these “just right” thresholds is paramount for the development of drugs with tolerable on-target dose-limiting toxicity. One potential limitation of our study is the low number of experimental repeats that prevented us from performing more detailed statistical analyses.

In conclusion, we present a murine in vivo model based on a simple binomial readout to test the activity of putative WNT antagonists, thereby providing a mammalian development-based alternative to the popular Xenopus axis duplication assay that has been used for over 30 years to study the WNT pathway and assess activities of its signalling antagonists [44,45,46].

## Figures and Tables

**Table 1:** WNT inhibitor treatment durations of pregnant *Apc^flox/flox^* mice and observed outcomes.

Inhibitor	Treatment	Outcome
None	NA	All progeny *Apc^flox/+^* (15/15, born alive, 5 litters)
G007-LK	E5–14	Complete abortion (no embryonic tissue detected at E15; 1 pregnancy)
G007-LK	E2–3	Complete abortion (no embryonic tissue detected at E15; 1 pregnancy)
G007-LK	E5–10	Complete abortion (no embryonic tissue detected at E15; 1 pregnancy)
G007-LK	E4–7	Complete abortion (no embryonic tissue detected at E15; 1 pregnancy)
G007-LK	E5–6	Deformed, non-viable embryos (3/3; E15, 1 litter, genotypes not determined)
G007-LK	E5–7	Rescue of headless phenotype in subset (5/8) of *Apc^min/flox^* embryos (E15, 2 litters, 17 embryos in total: 9 *Apc^flox/+^*, 8 *Apc^min/flox^*)
G007-LK	E5–7 & E9–10	Complete abortion (no embryonic tissue detected at E15; 1 pregnancy)
G007-LK	E5–7	Rescue of headless phenotype in subset (8/11) of born pups (3 litters, 19 pups in total: 8 *Apc^flox/+^*, 11 *Apc^min/flox^*)
Pyrvinium	E5–7	Rescue of headless phenotype in subset (1/3) of born pups (1 litter, 4 pups in total: 1 *Apc^flox/+^*, 3 *Apc^min/flox^*)

**Table 2:** Breakdown of head phenotypes of born *Apc^flox/+^* and *Apc^min/flox^* mice.

		Born Alive			Stillborn	
Drug	Genotype	Two Eyes	One Eye	No Eye	Cranial Defects	Litters (n)
none	*flox/+*	15	0	0	0	5
none	*min/flox*	0	0	0	0	
G007-LK	*flox/+*	7	1	0	0	3
G007-LK	*min/flox*	0	3	5	3	
Pyrvinium	*flox/+*	1	0	0	0	1
Pyrvinium	*min/flox*	0	0	1	2	

**Table 3:** Chi^2^ test of mice born with anophthalmia after treatment with WNT antagonists.

Treatment	Anophthalmia	Normal	Total
WNT antagonist	10	8	18
None	0	15	15
Total	10	23	33

Fisher’s exact test: *p* = 0.0003.

**Table 4:** Two independent sample studies with dichotomous endpoints.

Study Parameters	
Anophthalmia incidence (sporadic), group 1	4.3%
Anophthalmia incidence (treatment-induced), group 2	55.6%
Alpha	0.05
Beta	0.2
Power	0.8

## Data Availability

The data presented in this study are available on request from the corresponding authors.

## Supplementary Materials

The following supporting information can be downloaded at: https://www.mdpi.com/article/10.3390/biomedicines11102719/s1, Figure S1: Administration of TNKSi attenuates WNT pathway activity in vivo and partially rescues cranial abnormalities in vivo; Figure S2: In vivo administration of pyrvinium pamoate partially rescues cranial abnormalities; Figure S3: Development of liver zonation defects and intestinal polyposis in 6-month-old *Apc^flox/flox^* and *Apc^min/flox^* mice; Figure S4: Different threshold requirements of canonical WNT/β-catenin signalling during embryonic development and disease.
