# Supplementary material for: A Mouse Model for the Rapid and Binomial Assessment of Putative WNT/β-Catenin Signalling Inhibitors
Source: Biomedicines. 2023 Oct 7;11(10):2719. doi: 10.3390/biomedicines11102719 (PMC10604108; doi:10.3390/biomedicines11102719)
Supplement: Supplementary file 1 [file biomedicines-11-02719-s001.zip › biomedicines-2621601-supplementary.pdf]

A Mouse Model for the Rapid and Binomial Assessment of Putative WNT/β-Catenin Signalling Inhibitors

Janson Tse [1], Ryan O'Keefe [1,2], Angela Rigopolous [2,3], Annalisa L. E. Carli [1,2], Jo Waaler [4,5], Stefan Krauss [4,5], Matthias Ernst [1,2] and Michael Buchert [1,2,*]

Supplementary Materials:

Figure S1: *Administration of TNKSi attenuates WNT pathway activity in vivo and partially rescues cranial abnormalities in vivo.*
Figure S2: *In vivo administration of parvinium pamoate partially rescues cranial abnormalities.*
Figure S3: *Development of liver zonation defects and intestinal polyposis in 6-month-old Apc$^{flox/flox}$ and Apc$^{min/flox}$ mice.*
Figure S4: *Different threshold requirements of canonical WNT/β-catenin signalling during embryonic development and disease.*

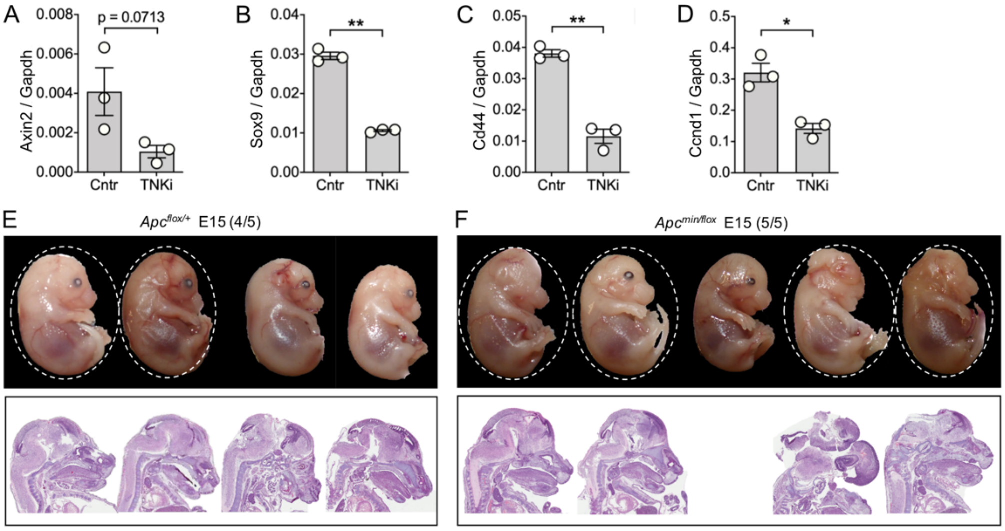

Figure S1. *Administration of TNKSi attenuates WNT pathway activity in vivo and partially rescues cranial abnormalities in vivo.* ***(A - D)*** *mRNA expression of downstream WNT target genes (Axin2, Sox9, Cd44 and Cyclin D1) in intestines of Apc$^{flox/flox}$ mice. Mice were treated with TNKSi or control for three consecutive days before euthanasia.* ***(E, F)*** *E15 embryos extracted from Apc$^{flox/flox}$ mice (top row) impregnated by Apc$^{min/+}$ male mice after treatment with G007-LK at embryonic days 5-7. Pictures were taken after extraction with accompanying H&E staining of sagittal sections (bottom row) to observe cranial features. Images show representative embryos of litter consisting of 5 Apc$^{flox/+}$ (**E**) and 5 Apc$^{min/flox}$ (**F**) embryos. Encircled embryos are the same as shown in Figure 1. Data are represented as Mean ± SEM. * p<0.05, ** p<0.01, unpaired t test.*

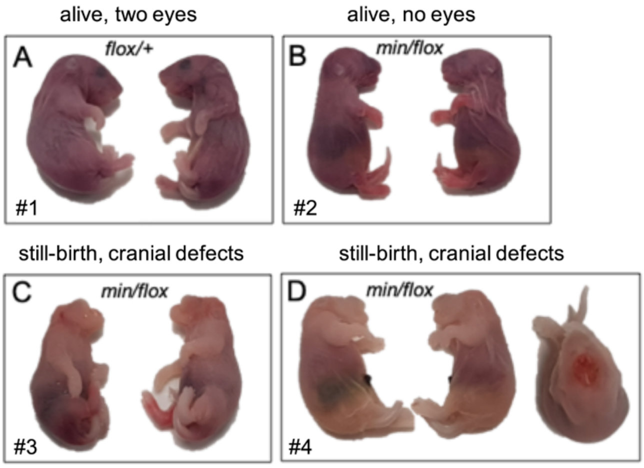

Figure S2. *In vivo administration of parvinium pamoate partially rescues cranial abnormalities. Pups, both alive (**A, B**) and still born (**C, D**), from Apc$^{flox/flox}$ female mice impregnated by Apc$^{min/+}$ males after treatment with parvinium pamoate at embryonic days 5-7. Pictures were taken at day of birth. Genotype and eye phenotype are as indicated.*

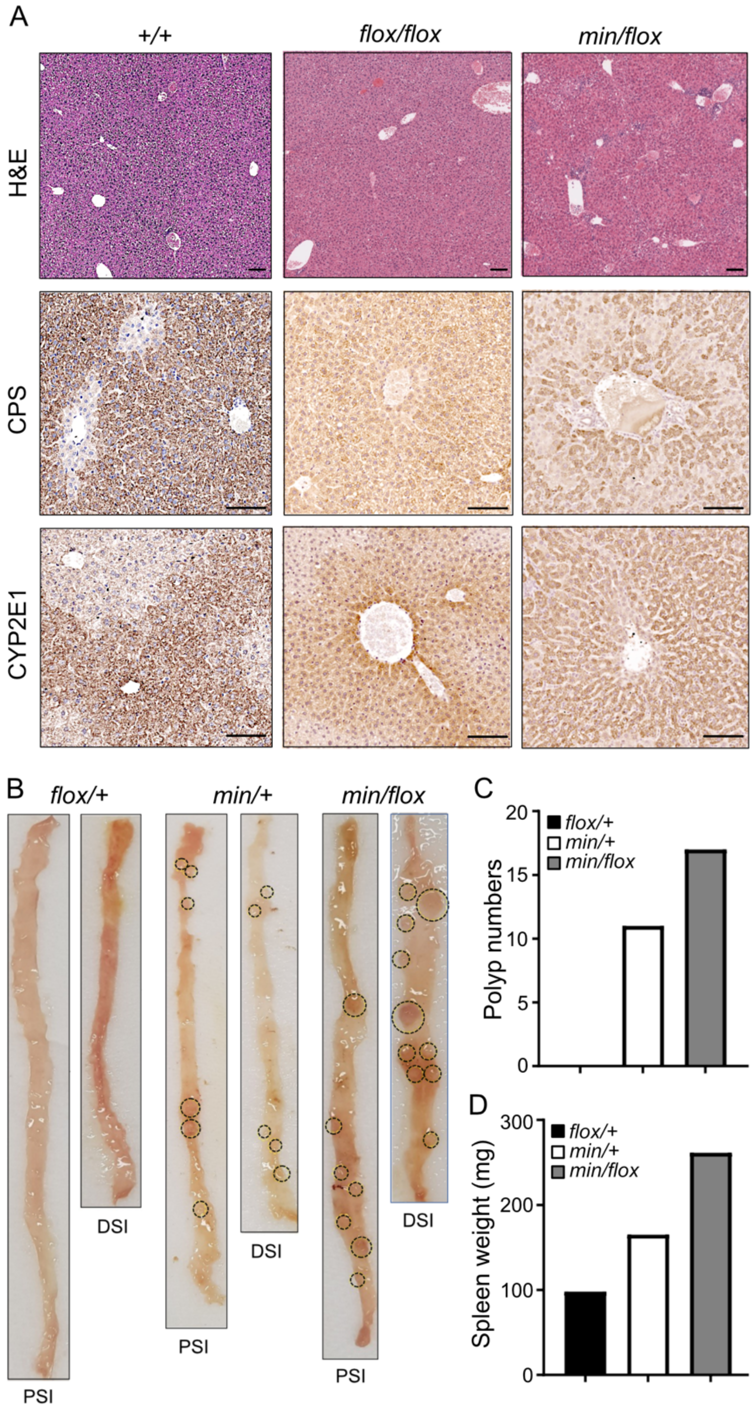

Figure S3. *Development of liver zonation defects and intestinal polyposis in 6-month-old Apc$^{flox/flox}$ and Apc$^{min/flox}$ mice. (**A**) Hematoxylin and eosin (H&E) and IHC stains of livers from 6-month-old wildtype (+/+), Apc$^{flox/flox}$ (flox/flox) and Apc$^{min/flox}$ (min/flox) mice. Sections were stained with H&E or with the indicated antibodies. (**B**) Images of longitudinally opened proximal (PSI) and distal (DSI) sections of the small intestine of Apc$^{flox/+}$, Apc$^{min/+}$ and Apc$^{min/flox}$ mice. Individual polyps are inside dashed circles. (**C**) Quantification of intestinal polyp numbers and spleen weights (**D**) of mice of the indicated genotypes (n=1). Scale bar = 100 μm.*

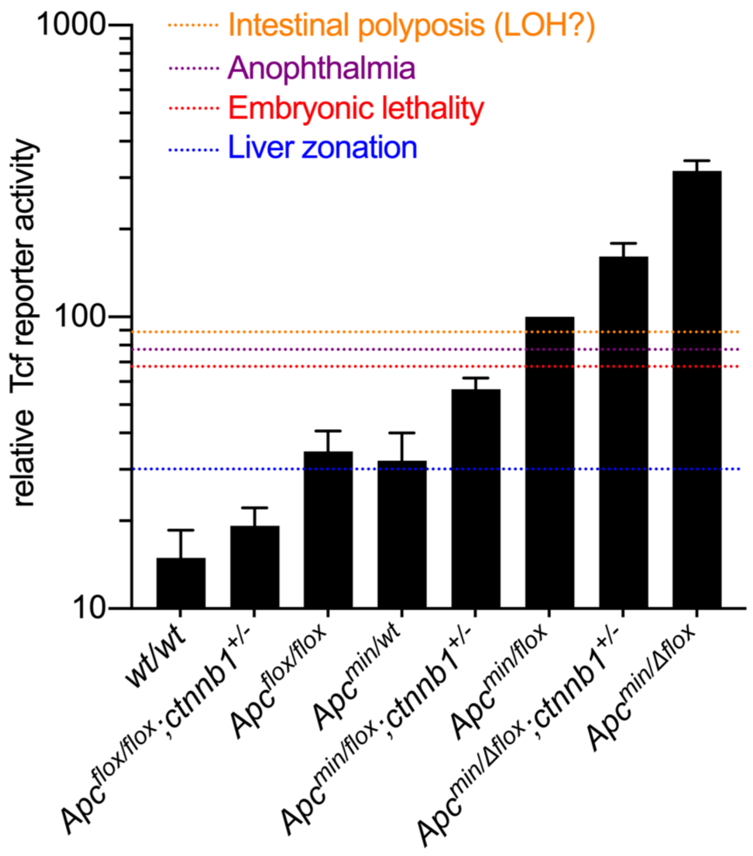

Figure S4. *Different threshold requirements of canonical WNT/β-catenin signalling during embryonic development and disease. TCF reporter activity is shown after stimulation of mouse embryonic fibroblasts (MEFs) of the indicated genotypes with sub-maximally active concentrations of WNT3a-conditioned medium. Horizontal dashed lines indicate the signalling thresholds predicted for the indicated phenotypes to occur in mice of the corresponding genotypes. 'LOH?' refers to the unknown requirement for 'loss of heterozygosity' of the 'flox' allele in intestinal stem cells of* $Apc^{min/flox}$ *mice for the initiation of polyp formation. Genotypes are as follows: wild-type (wt/wt),* $Apc^{min/+}$, $Apc^{flox/flox}$, $Apc^{min/flox}$, $Apc^{min/\Delta flox}$, $Apc^{flox/flox}$;*ctnnb1*$^{+/-}$, $Apc^{min/flox}$;*ctnnb1*$^{+/-}$, $Apc^{min/\Delta flox}$;*ctnnb1*$^{+/-}$. *Mean ± SEM. Data adapted from (Buchert et al., 2010).*
